# Impact on outcomes of ultra hypofractionation or hypofractionation regimens and proton or Xrays therapy in a phase III randomized controlled trial MC1635

**DOI:** 10.3389/fonc.2025.1557207

**Published:** 2025-08-06

**Authors:** Carlos Vargas, Kimberly Corbin, Lisa McGee, Robert Mutter, Heather Gunn, Michael Golafshar, Christopher Dodoo, Laura Vallow, Dean Shumway, Sameer Keole, Sean Park, Michele Halyard, Patricia Cronin, Barbara Pockaj

**Affiliations:** ^1^ Mayo Clinic Arizona, Scottsdale, AZ, United States; ^2^ Mayo Clinic, Rochester, MN, United States; ^3^ Mayo Clinic Florida, Jacksonville, FL, United States

**Keywords:** Phase III, randomized clinical trial, propective studies, breast cancer, proton radiation, ultrahypofractionation, hypofraction

## Introduction

The Early trialist cooperative group have shown a survival benefit for radiation in the setting of breast-conserving surgery (1). Overall, it reduced the risk of recurrence by 16% at 10 years and breast cancer-specific survival by 4% at 15 years. Different groups exhibit varying benefits, with larger advantages observed in LN+ patients, showing an absolute benefit of 9% in the 15-year breast cancer death risk. However, radiation therapy is not widely available in low to middle-income countries, with less than 10% of all available external beam machines (2). Even in the United States, access to oncologic care is restricted in rural communities, with up to 36% having to travel for cancer care. In addition, longer courses can be associated with higher financial toxicity, as relocation costs are higher and time off work may be prolonged.” (3–5). Regarding resource utilization and access, shorter radiation courses would help to decrease the equity gap (6).

Conventional fractionation for whole breast radiation was standard for many years; however, in recent years, moderate hypofractionation has been established as the standard of care option, based on non-inferior oncologic outcomes, with similar improvements in treatment toxicity. The UK breast standardization studies, START A and B, showed excellent cancer control and low rates of adverse events. More specifically, in START B, local-regional relapse (HR 0·77, 95% CI 0·51–1·16; p=0·21) was similar between groups; breast shrinkage, telangiectasia, and breast edema were significantly less common in the 40 Gy group compared to the 50 Gy group (7–9). Three phase III prospective controlled randomized trials have evaluated 5 fraction whole breast radiation (10–12). Two of them have evaluated consecutive treatments, while the third evaluated the use of weekly RT over 5 weeks (10). The UK FAST FORWARD trial evaluating 5 consecutive fractions compared with 15 fractions proved the safety and efficacy of this regimen. Overall, any breast cancer-related event was under 8%, with an overall breast cancer mortality under 4% for all groups., demonstrating non-inferiority for tumor control and safety for normal tissues effects, with no significant differences at 5 years of follow up for 26 Gy, compared with 40 Gy. Toxicity was not described as traditionally done in US trials, where adverse events based on the CTCAE are defined. However, looking at referrals for management of radiation-related adverse events, lymphedema ranged between 6.6% and 8.9% in the different groups, and referrals to other specialized services were under 3% in any of the 3 arms. Overall, patients reported outcomes (PROS) moderate or marked events were seen in about 11%-16%. Although longer term follow up is awaited, these data support the adoption of consecutive ultra-hypofractionation as a standard of care option for women with early stage breast cancer.

Herein, we report 5-year estimates for the phase III US trial comparing 5 vs 15 treatments for whole breast radiotherapy, using protons and photons. We also report PROS and adverse events for each modality and fractionation group. Differences between our study and the UK Fast Forward (12) trial include a lower dose of 25 Gy compared to 26 and 27 Gy. Simultaneous integrated boost was allowed, which helped maintain the number of treatments within 5 or 15 visits in the ultra-hypofractionated (UFH) or hypofractioned (HF) arm, respectively. Lastly, patients were allocated to different blocks based on modality, protons vs x-rays.

## Materials and methods

### Study design and participants

MC1635 was a randomized phase III controlled trial that accrued patients at multiple sites of a single institution and was performed after Institutional Review Board approval (IRB #16-008600). This trial was registered at the National Institutes of Health clinical trial registry (NCT03324802), available on ClinicalTrials.gov.

**Figure 1:**
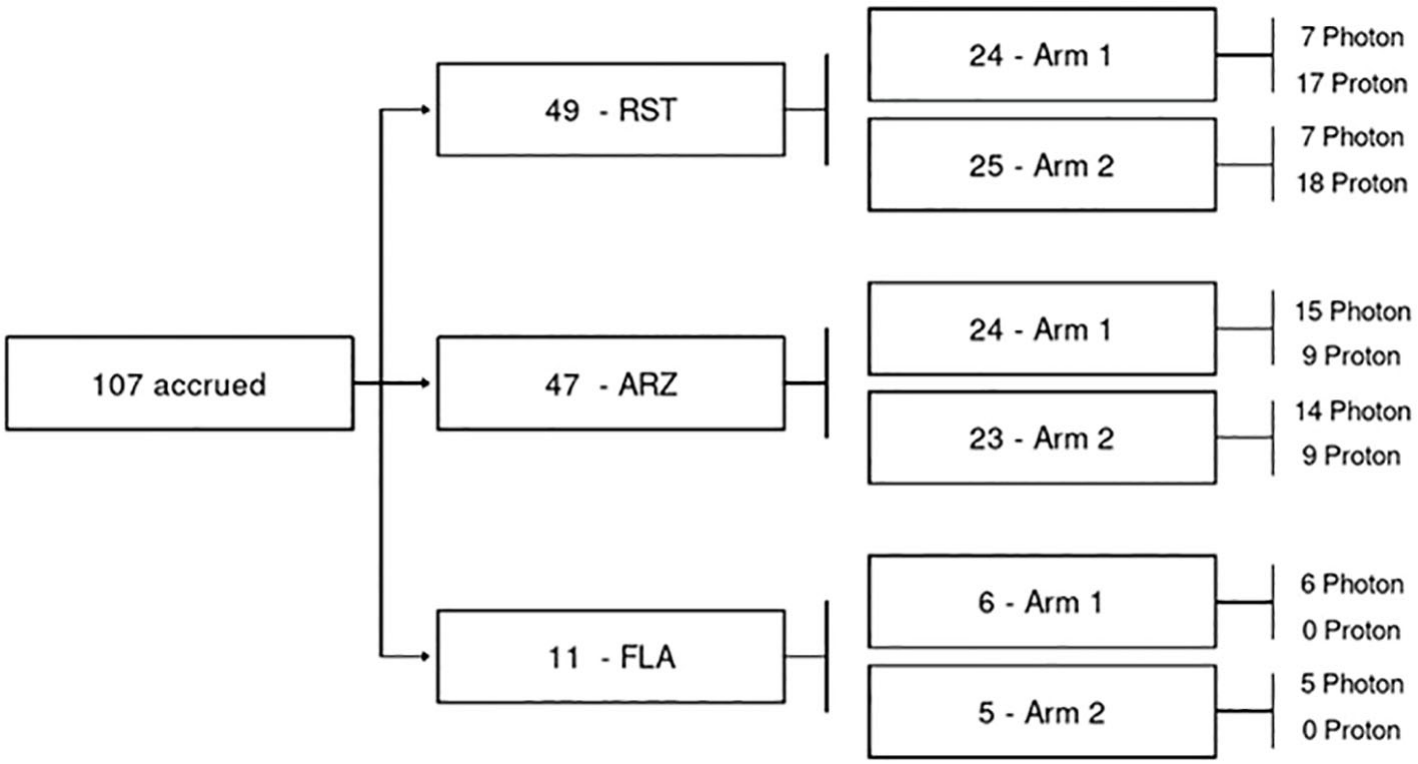
Consort diagram with treatment by modality.

Eligibility criteria included women aged 18 or older with AJCC 8th edition pathologic stage pT0–3 pN0–1 M0 breast cancer. Patients would have Eastern Cooperative Oncology Group (ECOG) performance status 0 to 2. Patients were randomized in a 1:1 fashion to hypofractionation (HF) consisting of 40 Gy in 15 fractions with an optional simultaneous integrated boost (SIB) to 48 Gy in 15 consecutive fractions (n= 54), or ultra-hypofractionation (UHF) consisting of 25 Gy (RBE)/Gy in 5 consecutive daily fractions with an optional SIB to a total dose of 30 Gy (RBE)/Gy in 5 fractions (n = 53) (**Figure 1**). The UHF dose used in the current trial was based on the work of Qi et al., who developed a model using data from large, randomized trials to estimate the α/β of breast cancer. Qi and colleagues estimated the α/β of breast cancer to be 2.88 (0.75-5.01) (13). Therefore, based on this work, 25 Gy in 5 fractions was selected as the ultra-hypofractionated dose/fractionation scheme as the radiobiological equivalent to 50 Gy in 25 fractions.

Exclusion criteria included patients with severe active co-morbid systemic illnesses or another severe concurrent disease. Other exclusion criteria included active systemic lupus, scleroderma, and prior ipsilateral chest wall irradiation.

### Treatment procedures

Radiation was delivered to the whole breast without nodal irradiation after breast-conserving surgery. Breast volumes were defined by the treating radiation oncologist, and in general, it was suggested to include the breast gland volume, usually defined laterally by the lateral internal thoracic vessels, medially the perforating branches of the internal thoracic vessels (aka internal mammary vessels). Superiorly, usually the breast tissue will be under the level head of the clavicle and inferiorly stopping before the curvature of the breast met the chest wall (14, 15). For left-sided cases, the heart was completely blocked based on a deep inspiration breath hold scan for x-rays. We also used a liver block when needed, and tangents were angled as needed to cover adequately the lumpectomy area while minimizing lung dose. For the boost volume, we usually included the seroma and tumor bed clips within our CTV. An additional 5mm margin limited 5 mm from the skin was used to create the PTV. All breast tissue was limited to 5 mm from the skin as well. Proton treatments were done with spot scanned anterior fields without breath-hold. Treatment could be delivered with photons or protons. Dosimetric plans were optimized on each arm with the following dose distributions: minimum dose received by 95% of breast CTV ≥95% of prescription, and breast CTV max dose <107% of prescription. Normal tissue constraints included: heart max dose ≤25% of prescription for protons and ≤33% of prescription for photons, heart mean dose <0.1 Gy for protons and <1 Gy for photons, and ipsilateral lung V50% ≤10% of prescription for both protons and photons. According to institutional guidelines, daily image guidance was performed with surface guidance and kV or CBCT. The principal investigator centrally reviewed the first two treatment plans per site.

### Study outcomes

The study’s primary objective was to determine the complication rate of 5-fraction versus 15- fraction whole breast radiotherapy, evaluating both regimens with or without a boost as well as photon versus proton modality. Complications were defined as one or more of the following parameters: Grade 3 or higher late adverse event according to CTCAE version 4, or deterioration of cosmesis from excellent/good to fair/poor, or from fair to poor. Secondary endpoints included acute and late toxicities, five-year locoregional control, disease-free survival, and overall survival. Cosmesis was assessed by the treating physician and classified according to the Harvard Cosmesis Scale as follows: 1) Excellent: Treated breast nearly identical to untreated breast; 2) Good: Treated breast slightly different than untreated; 3) Fair: Obvious difference in the size and shape of the treated breast and the change involves one-quarter or less of the breast; 4) Poor: Marked change in appearance or shape involving more than one-quarter of the breast, or the treated breast has severe sequelae from radiation therapy. Quality of life (QoL) was assessed according to the Patient-Reported Outcomes Measurement Information System (PROMIS), overall Linear Analogue Self-Assessment (LASA), and adapted Breast Cancer Treatment Outcome Scale (BCTOS). The LASA was scored using a scale of 0 to 10 for domains such as pain, fatigue, and overall quality of life. Toxicities, cosmesis, and QoL were assessed at baseline, end of treatment (EOT), 3 months, 1 year, 2 years, and 3 years from irradiation, while QoL was also assessed at 6 months. The average change from baseline was assessed over time. Change from baseline was adjusted so that >0 shows better outcomes (better QoL, less pain, etc.), while <0 shows worse outcomes.

### Statistical analysis

Patients were dynamically randomized between UHF and HF radiation and stratified by modality to enable evaluation of outcomes by modality. The current analysis evaluates results based on treatment modality —X-rays or protons— an exploratory endpoint of the study. ANOVA models were used to compare change score differences in QoL and chi-square tests were used to compare categorical outcomes (e.g., physician-scored toxicity) at baseline, EOT, 3 months, 6 months, 1 year, and years two and three following radiation delivery at each time interval. This analysis reported physician-scored toxicity and patient-reported QoL outcomes of breast cancer patients receiving standard-of-care HF or UHF adjuvant radiation therapy to the whole breast evaluating x-rays and protons. Differences QoL or physician-scored toxicity at baseline, EOT, 3 months, 6 months, 1 year, and years two and three following radiation delivery were analyzed at each time interval. All statistical tests performed were 2-sided with an *α* level of 0.05, and these analyses were performed using SAS version 9.4 (SAS Institute, Cary, NC). Patient blocks for protons and X-rays were available, thus comparisons between 5 and 15 treatments within a modality are more statistically appropriate than comparisons between modalities, i.e., X-rays vs. protons (**Figure 1**). Differences between groups were calculated and reported with confidence intervals.

## Results

From March 28, 2018, to February 11, 2020, 107 patients were enrolled and randomized to HF (15 fractions, n = 54, x-rays 28 and protons 26) or UHF (5 fractions, n = 53, x-rays in 26 and protons in 27) (**Figure 1**). The median follow-up was 51 months. Patient and disease characteristics are summarized in **Table 1**. Left breast cases were more commonly treated than right, and although most cases were HER2-negative and ER-positive, HER2-positive distribution was not balanced between groups. All other patient characteristics were balanced between all groups. Treatment characteristics are listed in **Table 2**. The radiation modality was spot scanning proton therapy (n = 54) or 3D conformal photon therapy (n = 53), with equal distribution in both arms. A simultaneous integrated boost (SIB) was used in 15 (28%) and 23 (43%) HF and UHF arms patients, respectively. Deep inspiration breath hold (DIBH) was used with 3D conformal x-rays for left-sided patients; no proton cases were treated with DIBH. A larger proportion of patients treated with protons received a boost than with x-rays. Only one case did not receive the protocol-specified dose and was treated to a slightly lower dose in the 5-treatment arm of 24.02Gy. All other patient treatment characteristics were well balanced between protons and x-rays.

**Table 1:** Patients’ characteristics for all groups.

	Photon		Proton			
	5 Fraction (N=26)	Conventional (N=28)	5 Fraction (N=27)	Conventional (N=26)	Total (N=107)	p value
Age at RT start						0.705^2^
Mean (SD)	62.0 (9.5)	61.6 (7.0)	63.7 (9.3)	63.8 (8.5)	62.8 (8.5)	
Median	62.8	61.6	65.6	67.0	64.0	
Range	43.7 - 77.5	45.9 - 75.1	45.8 - 81.7	41.1 - 78.1	41.1 - 81.7	
Race						0.544^1^
American Indian or Alaska Native	0 (0.0%)	1 (3.6%)	1 (3.7%)	0 (0.0%)	2 (1.9%)	
Black or African American	1 (3.8%)	1 (3.6%)	0 (0.0%)	0 (0.0%)	2 (1.9%)	
White	23 (88.5%)	24 (85.7%)	26 (96.3%)	26 (100.0%)	99 (92.5%)	
Unknown	2 (7.7%)	1 (3.6%)	0 (0.0%)	0 (0.0%)	3 (2.8%)	
Choose not to Disclose	0 (0.0%)	1 (3.6%)	0 (0.0%)	0 (0.0%)	1 (0.9%)	
Ethnicity						0.067^1^
Hispanic or Latino	3 (11.5%)	1 (3.6%)	0 (0.0%)	1 (3.8%)	5 (4.7%)	
Not Hispanic or Latino	21 (80.8%)	23 (82.1%)	27 (100.0%)	25 (96.2%)	96 (89.7%)	
Unknown or not reported	2 (7.7%)	4 (14.3%)	0 (0.0%)	0 (0.0%)	6 (5.6%)	
Study Breast						0.014^1^
Left	11 (42.3%)	10 (35.7%)	19 (70.4%)	18 (69.2%)	58 (54.2%)	
Right	15 (57.7%)	18 (64.3%)	8 (29.6%)	8 (30.8%)	49 (45.8%)	
Multiple Lesions						0.285^1^
Yes	6 (23.1%)	2 (7.1%)	6 (22.2%)	3 (11.5%)	17 (15.9%)	
No	20 (76.9%)	26 (92.9%)	21 (77.8%)	23 (88.5%)	90 (84.1%)	
Histologic Type						0.634^1^
Infiltrating ductal	15 (57.7%)	14 (50.0%)	16 (59.3%)	14 (53.8%)	59 (55.1%)	
Infiltrating lobular	6 (23.1%)	3 (10.7%)	5 (18.5%)	7 (26.9%)	21 (19.6%)	
Mixed infiltrating ductal and lobular	0 (0.0%)	1 (3.6%)	2 (7.4%)	1 (3.8%)	4 (3.7%)	
Mixed mammary carcinoma, NOS	1 (3.8%)	0 (0.0%)	1 (3.7%)	1 (3.8%)	3 (2.8%)	
DCIS	3 (11.5%)	8 (28.6%)	3 (11.1%)	3 (11.5%)	17 (15.9%)	
Other	1 (3.8%)	2 (7.1%)	0 (0.0%)	0 (0.0%)	3 (2.8%)	
Infiltrating ductal	15 (57.7%)	14 (50.0%)	16 (59.3%)	14 (53.8%)	59 (55.1%)	
Histologic Grade						0.172^1^
Grade I	12 (46.2%)	7 (25.0%)	5 (18.5%)	9 (34.6%)	33 (30.8%)	
Grade II	10 (38.5%)	12 (42.9%)	10 (37.0%)	12 (46.2%)	44 (41.1%)	
Grade III	4 (15.4%)	9 (32.1%)	12 (44.4%)	5 (19.2%)	30 (28.0%)	
Stage						0.198^1^
0	3 (11.5%)	10 (35.7%)	8 (29.6%)	5 (19.2%)	26 (24.3%)	
IA	16 (61.5%)	14 (50.0%)	14 (51.9%)	15 (57.7%)	59 (55.1%)	
IB	5 (19.2%)	4 (14.3%)	1 (3.7%)	3 (11.5%)	13 (12.1%)	
IIA	2 (7.7%)	0 (0.0%)	2 (7.4%)	3 (11.5%)	7 (6.5%)	
IIB	0 (0.0%)	0 (0.0%)	2 (7.4%)	0 (0.0%)	2 (1.9%)	
Lymph Nodes						0.072^1^
N0	23 (88.5%)	28 (100.0%)	26 (96.3%)	25 (96.2%)	102 (95.3%)	
N1	3 (11.5%)	0 (0.0%)	0 (0.0%)	0 (0.0%)	3 (2.8%)	
N1mic	0 (0.0%)	0 (0.0%)	1 (3.7%)	1 (3.8%)	2 (1.9%)	
Clinical M Stage						0.991^3^
M0	26 (100.0%)	28 (100.0%)	27 (100.0%)	26 (100.0%)	107 (100.0%)	
Tumor Size						0.378^1^
0-2 cm	23 (88.5%)	20 (71.4%)	23 (85.2%)	20 (76.9%)	86 (80.4%)	
>2-5 cm	3 (11.5%)	8 (28.6%)	4 (14.8%)	6 (23.1%)	21 (19.6%)	
Has the patient had any (other) prior cancer diagnosed?						0.112^1^
Yes	1 (3.8%)	8 (28.6%)	5 (18.5%)	4 (15.4%)	18 (16.8%)	
No	25 (96.2%)	20 (71.4%)	22 (81.5%)	22 (84.6%)	89 (83.2%)	
(If yes), site of prior cancer						0.315^1^
Basal/Squamous Cell Carcinoma	1 (100.0%)	1 (12.5%)	4 (80.0%)	1 (25.0%)	7 (38.9%)	
Endometrial carcinoma	0 (0.0%)	1 (12.5%)	0 (0.0%)	0 (0.0%)	1 (5.6%)	
Left Breast	0 (0.0%)	1 (12.5%)	1 (20.0%)	0 (0.0%)	2 (11.1%)	
Lymphoma	0 (0.0%)	2 (25.0%)	0 (0.0%)	0 (0.0%)	2 (11.1%)	
Melanoma	0 (0.0%)	1 (12.5%)	0 (0.0%)	1 (25.0%)	2 (11.1%)	
Right Breast	0 (0.0%)	2 (25.0%)	0 (0.0%)	0 (0.0%)	2 (11.1%)	
Thyroid	0 (0.0%)	0 (0.0%)	0 (0.0%)	2 (50.0%)	2 (11.1%)	
Weight						0.837^2^
Mean (SD)	79.3 (20.7)	76.9 (14.7)	75.4 (15.8)	75.8 (16.3)	76.8 (16.8)	
Median	72.5	74.6	72.5	75.0	73.6	
Range	53.1 - 143.0	56.5 - 117.0	52.1 - 120.6	49.6 - 120.2	49.6 - 143.0	
Height						0.448^2^
Mean (SD)	164.6 (7.0)	164.3 (6.8)	162.7 (6.5)	162.1 (5.9)	163.5 (6.6)	
Median	165.0	166.0	162.0	163.0	163.4	
Range	148.0 - 180.0	147.0 - 174.8	150.0 - 173.0	147.9 - 174.0	147.0 - 180.0	
ER % cells stained positive at diagnosis (Range)						0.308^1^
<10%	1 (5.6%)	4 (20.0%)	0 (0.0%)	2 (14.3%)	7 (10.9%)	
>90%	9 (50.0%)	8 (40.0%)	9 (75.0%)	9 (64.3%)	35 (54.7%)	
10-90%	8 (44.4%)	8 (40.0%)	3 (25.0%)	3 (21.4%)	22 (34.4%)	
HER2 gene expression (at diagnosis)						0.064^1^
Amplified	3 (11.5%)	0 (0.0%)	2 (7.4%)	1 (3.8%)	6 (5.7%)	
Was Ki67 performed?						0.408^1^
Yes	9 (34.6%)	7 (25.0%)	11 (40.7%)	12 (46.2%)	39 (36.4%)	
No	17 (65.4%)	21 (75.0%)	16 (59.3%)	14 (53.8%)	68 (63.6%)	
(If yes) Ki67 score						0.183^2^
Mean (SD)	22.6 (27.8)	8.4 (4.5)	22.8 (20.3)	11.1 (7.5)	16.4 (17.8)	
Median	12.0	7.0	15.0	10.0	10.0	
Range	0.4 - 87.0	5.0 - 17.6	1.0 - 60.0	2.0 - 25.0	0.4 - 87.0	
Did patient receive neoadjuvant therapy?						0.467^1^
Yes	2 (7.7%)	1 (3.6%)	2 (7.4%)	4 (15.4%)	9 (8.4%)	
No	24 (92.3%)	27 (96.4%)	25 (92.6%)	22 (84.6%)	98 (91.6%)	
Was anti-HER2 therapy administered?						0.872^1^
Yes	2 (7.7%)	1 (3.6%)	1 (3.7%)	1 (3.8%)	5 (4.7%)	
No	24 (92.3%)	27 (96.4%)	26 (96.3%)	25 (96.2%)	102 (95.3%)	
Did the patient receive neoadjuvant hormonal/endocrine therapy?						0.288^1^
Yes	3 (11.5%)	1 (3.6%)	0 (0.0%)	2 (7.7%)	6 (5.6%)	
No	23 (88.5%)	27 (96.4%)	27 (100.0%)	24 (92.3%)	101 (94.4%)	
Axillary surgery						0.206^1^
None	4 (15.4%)	10 (35.7%)	3 (11.1%)	5 (19.2%)	22 (20.6%)	
Axillary node dissection	0 (0.0%)	0 (0.0%)	0 (0.0%)	2 (7.7%)	2 (1.9%)	
Sentinel node biopsy followed by axillary node dissection	2 (7.7%)	2 (7.1%)	1 (3.7%)	1 (3.8%)	6 (5.6%)	
Sentinel lymph node biopsy only	18 (69.2%)	15 (53.6%)	23 (85.2%)	17 (65.4%)	73 (68.2%)	
Other	2 (7.7%)	1 (3.6%)	0 (0.0%)	1 (3.8%)	4 (3.7%)	
Bilateral						0.393^1^
Yes	0 (0.0%)	0 (0.0%)	1 (3.7%)	0 (0.0%)	1 (0.9%)	
No	26 (100.0%)	28 (100.0%)	26 (96.3%)	26 (100.0%)	106 (99.1%)	
Multicentric or multifocal disease?						0.310^1^
Yes	2 (7.7%)	1 (3.6%)	5 (18.5%)	3 (11.5%)	11 (10.3%)	
No	24 (92.3%)	27 (96.4%)	22 (81.5%)	23 (88.5%)	96 (89.7%)	
LVSI						0.271^1^
Yes	1 (4.0%)	0 (0.0%)	3 (11.1%)	1 (3.8%)	5 (4.7%)	
No	24 (96.0%)	28 (100.0%)	24 (88.9%)	25 (96.2%)	101 (95.3%)	
Missing	1	0	0	0	1	
DCIS present						0.308^1^
Yes	7 (26.9%)	10 (35.7%)	14 (51.9%)	10 (38.5%)	41 (38.3%)	
No	19 (73.1%)	18 (64.3%)	13 (48.1%)	16 (61.5%)	66 (61.7%)	

**Table 2:** Treatment characteristics for all patients.

	Photon		Proton			
	5 Fraction (N=26)	Conventional (N=28)	5 Fraction (N=27)	Conventional (N=26)	Total (N=107)	p value
Was chest wall motion control used -Deep Inspiration Breath Hold (DIBH)?						< 0.001^1^
Yes	11 (42.3%)	11 (39.3%)	0 (0.0%)	0 (0.0%)	22 (20.6%)	
No	15 (57.7%)	17 (60.7%)	27 (100.0%)	26 (100.0%)	85 (79.4%)	
Were there any unscheduled interruptions in radiation therapy?						0.416^1^
Yes	0 (0.0%)	1 (3.6%)	0 (0.0%)	0 (0.0%)	1 (0.9%)	
No	26 (100.0%)	27 (96.4%)	27 (100.0%)	26 (100.0%)	106 (99.1%)	
Total dose (not including boost) (cGy)						< 0.001^2^
Mean (SD)	2500.0 (0.0)	3947.6 (302.7)	2500.0 (0.0)	4005.0 (0.0)	3244.5 (756.9)	
Median	2500.0	4005.0	2500.0	4005.0	2500.0	
Range	2500.0 - 2500.0	2403.0 - 4005.0	2500.0 - 2500.0	4005.0 - 4005.0	2403.0 - 4005.0	
Did the patient receive a breast boost?						< 0.001^1^
Yes	6 (23.1%)	4 (14.3%)	17 (63.0%)	11 (42.3%)	38 (35.5%)	
No	20 (76.9%)	24 (85.7%)	10 (37.0%)	15 (57.7%)	69 (64.5%)	
(If yes) , RT total dose to boost field (cGy)						< 0.001^2^
Mean (SD)	3000.0 (0.0)	4800.0 (0.0)	3000.0 (0.0)	4800.0 (0.0)	3710.5 (891.6)	
Median	3000.0	4800.0	3000.0	4800.0	3000.0	
Range	3000.0 - 3000.0	4800.0 - 4800.0	3000.0 - 3000.0	4800.0 - 4800.0	3000.0 - 4800.0	
(If yes) , total number of fractions to boost field						< 0.001^2^
Mean (SD)	5.0 (0.0)	15.0 (0.0)	5.0 (0.0)	15.0 (0.0)	8.9 (5.0)	
Median	5.0	15.0	5.0	15.0	5.0	
Range	5.0 - 5.0	15.0 - 15.0	5.0 - 5.0	15.0 - 15.0	5.0 - 15.0	
Did the patient receive the protocol-specified dose?						0.416^1^
Yes	26 (100.0%)	27 (96.4%)	27 (100.0%)	26 (100.0%)	106 (99.1%)	
No	0 (0.0%)	1 (3.6%)	0 (0.0%)	0 (0.0%)	1 (0.9%)	

### Treatment toxicities

At the End of Treatment (EOT) assessment, grade 2 radiation dermatitis was experienced by four patients (7.4%) in the HF arm and by one patient (1.9%) in the UHF arm (p=0.36). At the one-year post- treatment assessment, one patient treated in the UHF arm demonstrated grade 2 superficial soft tissue fibrosis. When looking at the different groups, a total of 5 grade 2 events occurred: one treated with UHF protons (dermatitis n=1) and 4 patients treated with MHF protons (soft tissue fibrosis n=1 and dermatitis n=3). When comparing the four groups overall rates of grade 2 toxicities were for UHF (x-rays 0%, protons 3.7%) compared to HF (x-rays 0%, protons 15.4%), with a p-value of 0.01. The largest difference between groups corresponding to the UHF vs HF proton arms, p=0.19. More importantly, no grade 3 treatment-related events were seen (p=0.99), as shown in **Table 3**.

**Table 3:** Treatment related toxicities per group for all patients.

	Photon 5 Fraction (N=26)	Photon Conventional (N=28)	Proton 5 Fraction (N=27)	Proton Conventional (N=26)	Total (N=107)	p-value
All Grade 2+						0.01
Yes	0 (0.0%)	0 (0.0%)	1 (3.7%)	4 (15.4%)	5 (4.7%)	
No	26 (100.0%)	28 (100.0%)	26 (96.3%)	22 (84.6%)	102 (95.3%)	
Grade 2+ (acute)						0.09
Yes	0 (0.0%)	0 (0.0%)	1 (3.7%)	3 (11.5%)	4 (3.7%)	
No	26 (100.0%)	28 (100.0%)	26 (96.3%)	23 (88.5%)	103 (96.3%)	
Grade 2+ (chronic)						0.37
Yes	0 (0.0%)	0 (0.0%)	0 (0.0%)	1 (3.8%)	1 (0.9%)	
No	26 (100.0%)	28 (100.0%)	27 (100.0%)	25 (96.2%)	106 (99.1%)	
All Grade 3+						0.99
No	26 (100.0%)	28 (100.0%)	27 (100.0%)	26 (100.0%)	107 (100.0%)	

### Breast cosmesis

Excellent/good cosmesis was predominantly observed prior to radiation across all four groups: UHF x-rays (100%), UHF protons (96.3%), HF x-rays (100%), and HF protons (96.2%), p-value of 0.25. Examination of medical photographs by physicians revealed a single instance of cosmesis deterioration from excellent/good to fair/poor in a patient treated with protons in the HF arm at 3 years, as per the Harvard Cosmesis Scale. Nonetheless, results remained consistent across groups, with a p-value of 0.23.

### Quality of life metrics

**Figure 2:**
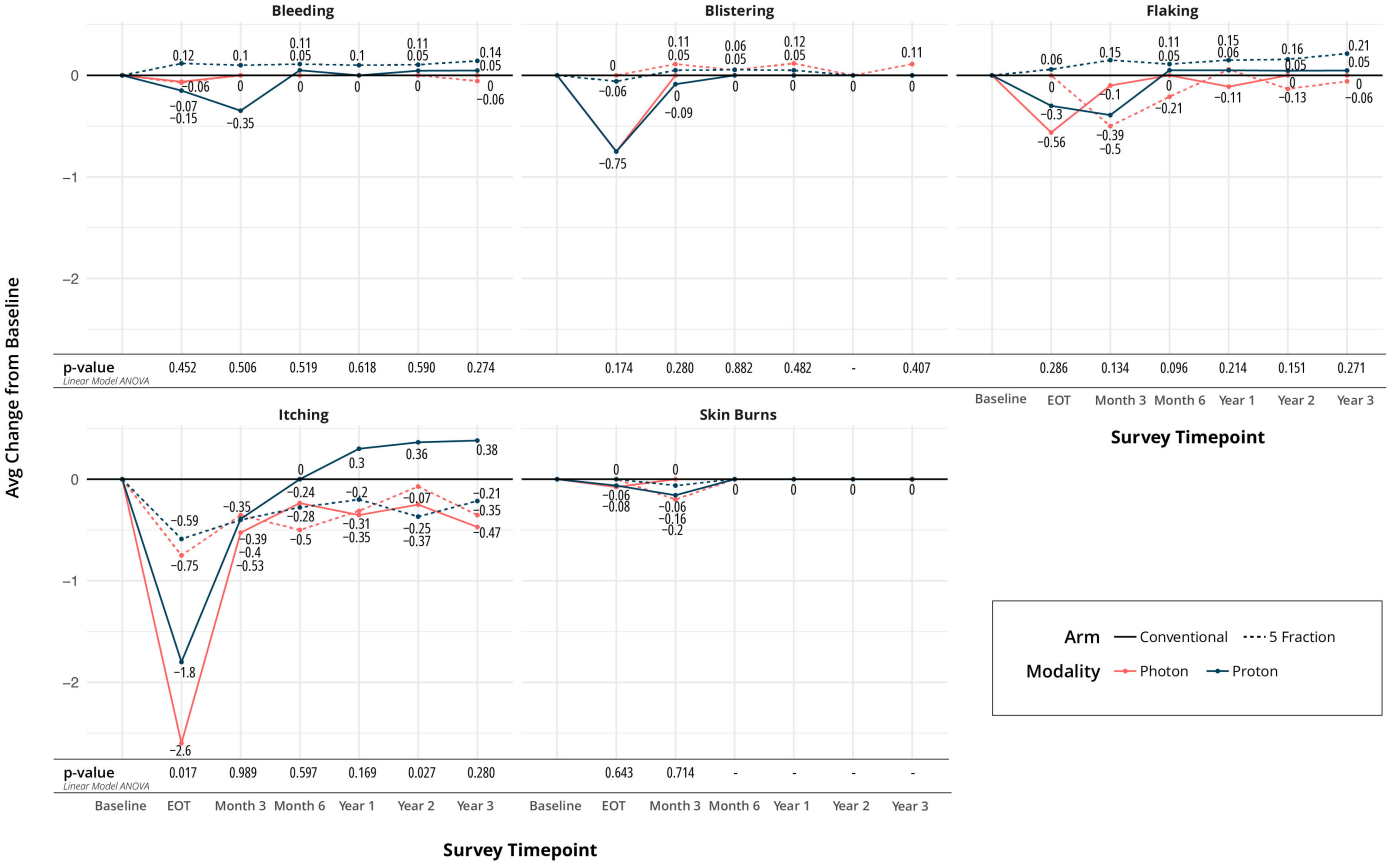
Changes from baseline in selected patient-reported skin-related metrics.

Most quality of life (QoL) metrics remained similar over time. There was no difference in the perception of shortness of breath, coughing, or swallowing at any time point between the four arms. Regarding bleeding, blistering, flaking, itching, and skin burns, only itching and skin burns showed a difference at the end of treatment. Minimal and higher skin burns were different between arms at end of treatment (EOT), favoring the UHF arms (p=0.002), as seen in **Figure 2A**. The largest difference was seen between UHF protons and HF protons in **Figure 2**. Itching at the EOT was also worse for the HF arms (p=0.017).

**Figure 3:**
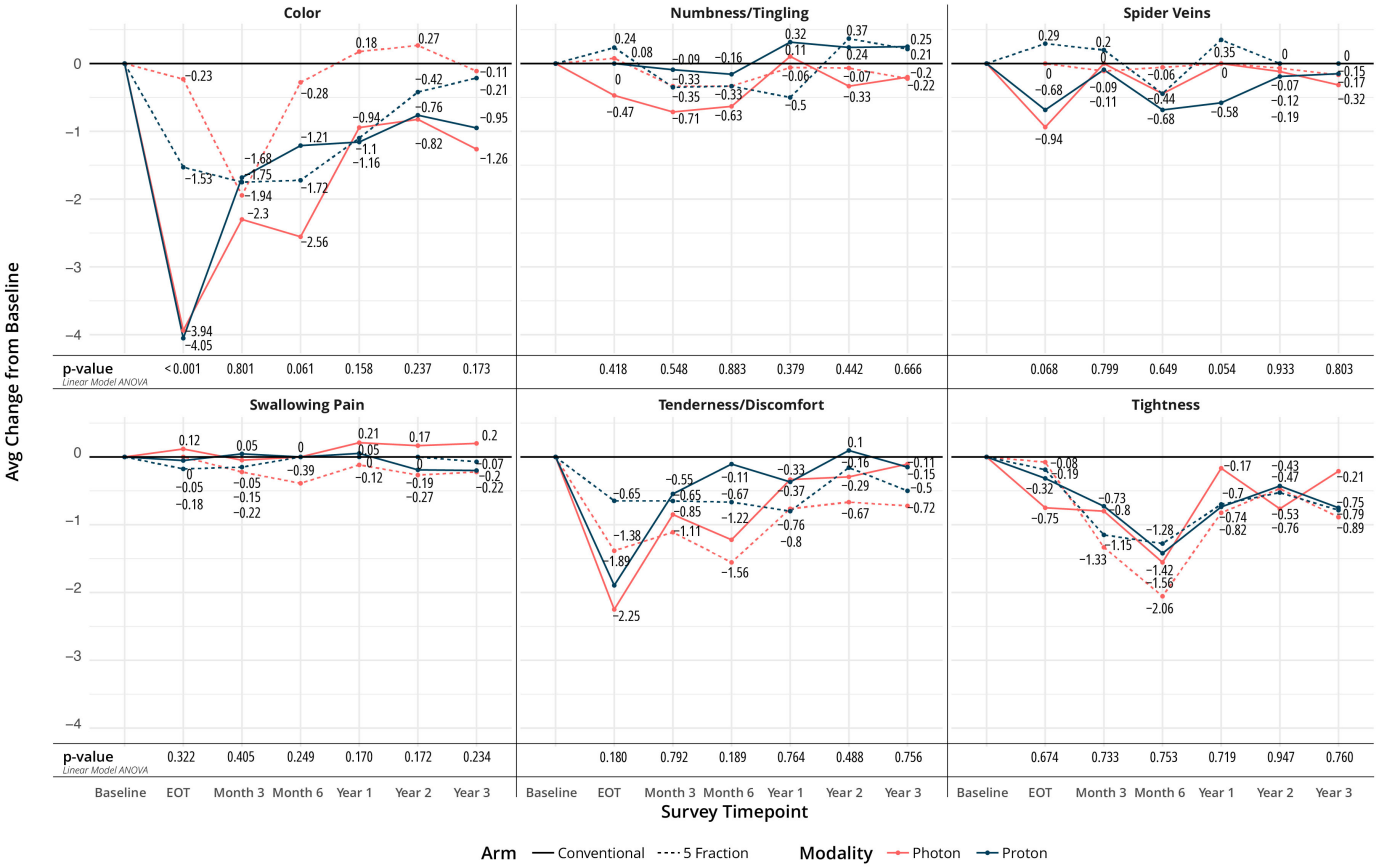
Changes from baseline in selected patient-reported breast-related metrics.

Regarding appearance and functioning, numbness, spider veins, swallowing pain, tenderness, and tightness did not show any difference between arms over time. However, color changes of the treated breast were different between groups at EOT and favored the UHF arms (p<0.001), **Figure 3**. Changes in overall quality of life, pain, and fatigue from baseline were not statistically different at EOT, 1-, 2-, and 3- year time points.

### 2-way ANOVA results of the interactions between modality and fractionation

The effect of UHF vs. HF on grade 2 toxicity was non-significant, while the effect of protons vs. x-rays reached significance, as shown in **Table 4**. However, when examining individual interactions between all possible combinations of modality (protons or x-rays) and fractionation (UHF or HF), we observed that the main effect was due to the increased toxicity seen with HF proton radiation compared to UHF or HF x-rays. Patients and physicians could choose protons or x-rays, leading to potential confounding variables affecting the outcomes of proton therapy and decreasing the value of a potential difference between modalities (protons vs. x-rays). Nonetheless, comparisons within modalities remain possible. These results suggested no effect of UHF vs. HF for x-rays but hinted at a benefit for the use of UHF for protons.

**Table 4:** 2-way ANOVA results for toxicity and QoL metrics with significant effects.

Outcome	Effect	p-value	Groups compared^a^	Difference in proportions	95% CI
Grade 2+	Arm main effect	N/A^b^	UHF vs. HF	-0.055	-0.134 – 0.024
	Modality main effect	N/A^b^	X-rays vs. Protons	-0.094	-0.173 – -0.016
	Arm × Modality interaction	N/A^b^	UHF-X vs. HF-X	N/A	N/A
			UHF-P vs. HF-P	-0.117	-0.273 – 0.039
			UHF-X vs. HF-P	-0.154	-0.293 – -0.015
			UHF-P vs. HF-X	0.037	-0.034 – 0.108
			UHF-X vs. UHF-P	-0.037	-0.108 – 0.034
			HF-X vs. HF-P	-0.154	-0.293 – -0.015
Outcome	Effect	p-value	Groups Compared	Difference in Means	95% CI
Skin burns EOT	Arm main effect	<0.001	UHF vs. HF	0.677	0.396 – 0.959
	Modality main effect	0.366	X-rays vs. Protons	0.150	-0.174 – 0.474
	Arm × Modality interaction	0.179	UHF-X vs. HF-X	0.466	0.049 – 0.883
			UHF-P vs. HF-P	0.849	0.472 – 1.226
			UHF-X vs. HF-P	0.786	0.387 – 1.185
			UHF-P vs. HF-X	0.529	0.133 – 0.926
			UHF-X vs. UHF-P	-0.063	-0.480 – 0.354
			HF-X vs. HF-P	0.319	-0.058 – 0.697
Itching EOT	Arm main effect	0.003	UHF vs. HF	1.488	0.523 – 2.452
	Modality main effect	0.331	X-rays vs. Protons	-0.535	-1.570 – 0.501
	Arm × Modality interaction	0.518	UHF-X vs. HF-X	1.850	0.356 – 3.344
			UHF-P vs. HF-P	1.212	-0.061 – 2.484
			UHF-X vs. HF-P	1.050	-0.359 – 2.459
			UHF-P vs. HF-X	2.012	0.645 – 3.378
			UHF-X vs. UHF-P	-0.162	-1.616 – 1.293
			HF-X vs. HF-P	-0.800	-2.118 – 0.518
Change in color EOT	Arm main effect	<0.001	UHF vs. HF	3.033	1.785 – 4.281
	Modality main effect	0.265	X-rays vs. Protons	0.585	-0.875 – 2.045
	Arm × Modality interaction	0.350	UHF-X vs. HF-X	3.707	1.832 – 5.581
			UHF-P vs. HF-P	2.523	0.847 – 4.199
			UHF-X vs. HF-P	3.822	2.015 – 5.629
			UHF-P vs. HF-X	2.408	0.660 – 4.157
			UHF-X vs. UHF-P	1.299	-0.551 – 3.148
			HF-X vs. HF-P	0.115	-1.588 – 1.818

^a^The difference between groups is always the first group listed minus the second group. ^b^Because of the low cell counts/no variability, a parameter could not be calculated.

The effect of UHF vs HF was significant for QOL over several different areas as seen in **Table 4**. However, modality (x-rays vs protons) did not show any significant interaction for any of the measured QOL metrics employed in the study.

### 5-year breast cancer outcomes

No invasive local failures have occurred, as well as no breast-related or overall deaths; our 5-year estimates for invasive local failures, breast cancer survival, and overall survival are 100%. A total of 4 breast cancer events have been observed, including 2 DCIS failures, one nodal regional failure, and one distant metastasis, for a 5-year disease-free survival estimate of 96% (95% CI:91-100% p-value=0.22).

## Discussion

The MC1635 randomized trial compared HF (40.05 Gy in 15 fractions) to UHF (25 Gy in 5 fractions) adjuvant whole breast radiation for patients with localized breast cancer not requiring nodal radiation. The current study showed small differences in grade 2 AEs, particularly an increase in grade 2 AEs with HF proton therapy. At the same time, UHF showed no difference in grade 2 AEs between x- rays and protons and demonstrated a consistent benefit for several QOL metrics. Both fractionation regimens and modalities yielded favorable cosmetic outcomes.

The UK Forward study compared 50 Gy over 5 weeks with weekly radiation to doses of 30 Gy or 28.5 Gy (10). Changes in breast appearance documented by photographs were done in 71% of patients and suggested a change in appearance (OR 1.64) with 30 Gy but no difference with 28.5 Gy when compared with 50 Gy over 5 weeks. This suggests a relatively low α/β for radiation-related breast visual changes (7, 13, 16). UK Fast Forward showed overall fewer adverse events in the breast with 26Gy than 27Gy compared to 40Gy over 3 weeks. These data also suggests that small changes in dose over 5 treatments can produce significant normal tissue effects. It also suggests a low α/β for breast cancer; only 11 events were seen with 10-year ipsilateral breast event rates of less than 2% in all arms.

Our study suggests that differences in normal tissue may be further reduced by an additional 1 Gy reduction in dose to 25 Gy over 5 treatments. In our study, UHF arms showed lower side effects grade 2 or higher than HF arms. In addition, PROs showed small but significant differences in tissue effect by the end of radiation among the four groups favoring the UHF arms (17–19). It is also noticeable that the UK studies found a difference in breast size (edema) moderate or marked and increased fibrosis; in our study, patients reported similar variations in breast size in the four groups. This is critically important as fewer treatments, especially if they are better tolerated, will improve access to care and reduce treatment costs (2, 20–23).

5-year estimates were encouraging; no invasive-breast recurrences, breast-related mortality, or overall mortality in the groups. Only 2 DCIS local failures were seen, accounting for less than 2% failure, similar to the UK studies. Two additional failures outside of the breast were observed, for a total of 4 breast cancer-related events, representing an estimated 3.7% total events that compare favorably with the Fast Forward study, which reported just under 8% total cancer events at 5 years. Our clinical information compares favorably with preoperative radiation studies suggesting that relative low hypofractionated doses such as 25Gy in 5 treatments are associated with good response rates (24). Response rates in preoperative studies further suggest that doses of 25Gy may be sufficient for the control of microscopic breast cancer residual disease (25–27).

In our study, we also examined the use of proton therapy. The current analysis aimed to investigate the effect of proton therapy for ultra-hypofractionated (UHF) and hypofractionated (HF) regimes. The study groups were balanced and stratified by modality to allow meaningful comparisons within modalities, specifically between UHF and HF protons. While the difference in Grade 2 adverse events (AEs) favoring UHF over HF protons was apparent, it was not statistically significant.

Additionally, several quality of life (QOL) metrics indicated an advantage of UHF for protons, with a significant difference observed in skin burns between the UHF and HF proton arms. This finding is noteworthy for the proton breast therapy community, as proton therapy has been associated with higher rates of skin burns in prior publications (28–33).

The increased skin burns in traditional proton therapy have been attributed the lack of superficial sparing effect of double scatter proton techniques. Efforts to reduce doses to normal tissues, particularly the lungs and heart, with proton therapy have led to higher doses to the skin with double scatter techniques. However, the implementation of scanning techniques allows for dose shaping away from the skin and within the breast clinical target volume. This approach spares both the skin and normal tissues at the distal edge of the target, resulting in reduced doses to the skin, chest wall, lung, and heart compared to traditional proton techniques. Previous studies have shown decreased mortality with lower heart doses (34–36).

Therefore, if UHF reduces skin burns in proton therapy, it may offer better-tolerated breast proton radiation treatments with decreased cardiac doses.

Our study showed no statistically significant difference in the deterioration of cosmesis from the provider’s perspective following treatment with protons or x-rays with both UHF and HF. Notably, no grade 3 acute or chronic toxicity has been reported in any of the four groups, underscoring the safety of ultra-hypofractionated RT and the use of different modalities, such as protons or X-rays.

This clinical trial has several limitations. Comparing two dose regimens and modalities with few events complicates *post-hoc* analysis and group comparisons. Including patients with either positive or negative lymph nodes, as well as those who underwent breast-conserving surgery or mastectomy, introduces heterogeneity that reduces internal consistency but may enhance real-world applicability. Patient-reported outcomes are subjective by nature, reflecting daily symptom perceptions. Variations in breast domain scores over time are influenced by personal perceptions, but reflecting relevant changes as perceived by the patient. Given the potential impact of a boost on adverse events, this will be evaluated in a separate manuscript. However, overall, fewer adverse events were observed with a UHF schedule. Additionally, a higher proportion of patients received a boost in the UHF group (43%) compared to the HF group (28%). Thus, it is possible that the effect of a boost on adverse events may not have been as pronounced. Ordering Ki-67 is not part of our standard practice, and as a result, the proportion of patients with available Ki-67 data was low. Although Ki-67 is associated with a higher proliferation rate, it is not an independent predictor of local control or survival. Since no invasive recurrences have been observed within the radiated area, it is unlikely that Ki-67 values would meaningfully differentiate local control rates within our cohort. An additional limitation is that skin changes typically peak about two weeks after the last x-ray or proton treatment, but patients were evaluated three months after treatment. Although physicians reported the peak toxicity during the 3-month evaluation, the patients’ medical records were reviewed for missed adverse events, and every ER visit and hospital admission was examined for possible relation to the treatment. It is possible, however, that some adverse events may have been missed between visits. However, this effect is likely lower in a study conducted within a relatively closed health system, where all health-related events can be more accurately evaluated, compared to multi-institutional phase III studies.

## Conclusions

Ultra-hypofractionated radiation of 25 Gy over 5 treatments showed excellent local control, leading to high cancer-specific and overall survival rates. UHF had low grade 2 adverse events and fewer mild or higher patient-reported events. Protons particularly benefited from UHF, with reduced grade 2 adverse events and improved QoL of mild severity or higher at the end of treatment.

## Data Availability

The datasets presented in this article are not readily available because The study was supported by clinical funds from the department of radiation oncology at Mayo Clinic and they are not for free distribution. Requests to access the datasets should be directed to carlos vargas vargas.carlos@mayo.edu.
